# Quality Control of Radix Astragali (The Root of *Astragalus membranaceus* var. *mongholicus*) Along Its Value Chains

**DOI:** 10.3389/fphar.2020.562376

**Published:** 2020-12-04

**Authors:** Yaqiong Bi, Haiying Bao, Chunhong Zhang, Ruyu Yao, Minhui Li

**Affiliations:** ^1^ Inner Mongolia Autonomous Region Academy of Traditional Medicine, Hohhot, China; ^2^ Jilin Agricultural University, Changchun, China; ^3^ Baotou Medical College, Baotou, China; ^4^ Inner Mongolia Engineering Research Center of the Planting and Development of Astragalus membranaceus of the Geoherbs, Baotou, China; ^5^ Institute of Medicinal Plant Development, Chinese Academy of Medical Sciences and Peking Union Medical College, Beijing, China

**Keywords:** radix astragali, daodi herb, value chain, quality, grade

## Abstract

Radix Astragali (RA), the root of *Astragalus membranaceus* var. *mongholicus* (Bunge) P.K. Hsiao, known as “Huangqi” in Chinese, has been used as a traditional herbal medicine or food in China for more than 2,000 years and is now consumed globally. Unfortunately, the increasing demand for RA has led to the overexploitation of its wild stock, as well as quality problems, including adulteration and contamination. Therefore, the sustainable cultivation of RA is urgently needed. In the present research, semi-structured interviews and key informant interviews were conducted, over a 2-year period, to collect data from stakeholders in the main production areas; moreover, a targeted chemical analysis-based quality assessment strategy was applied to understand the quality of RA. Accordingly, 10 different types of value chains (VCs) were identified in RA production; meanwhile, the contents of the main active ingredients (astragaloside and calycosin-7-O-*β*-D-glucoside) were analyzed by HPLC-ELSD-UV and the yield of medicinal material was demined and further analyzed using *k*-means clustering analysis. The results show that the tight relationship between quality of the RA and stakeholders’ revenues among the VCs, which reflects a more general trend in the production system. Over the past few decades, vertical coordination has emerged increasingly in VCs of RA, which leads to a more coherent traceability system and rigorous regulations in the supply chains. *Daodi* herbs can be considered to be a standard that is distinctive with good quality characteristics that emphasize the origins of the medicinal plants. We find that the suitability of geographical areas and vertical integration can improve the VCs of RA, which further contributes to its quality control, as well as its sustainable production.

## Introduction

Radix Astragali (RA), the root of *Astragalus membranaceus* var. *mongholicus* (Bunge) P.K. Hsiao or *A. membranaceus* (Fisch.) Bge. is often used in traditional Chinese medicine. It was classified as top grade in *Shennong Bencao Jing* (*Shennong’s Materia Medica Classic*), which means adverse side effects from the medicine material are uncommon and can be taken for a long time. It has been widely used in foods, teas, drinks, wines, cosmetics, and so on (Guo et al., 2018). In fact, according to the statistics, RA is used in more than 192 Chinese medicine prescriptions and over 1,045 kinds of health products (Cheng et al., 2019). In recent years, RA is also increasingly consumed by the global market beyond China. The global consumption of RA is vast, for example, 4,477.17 tons of RA have been exported from China in 2015 (Wang et al., 2018).

**FIGURE 1:**
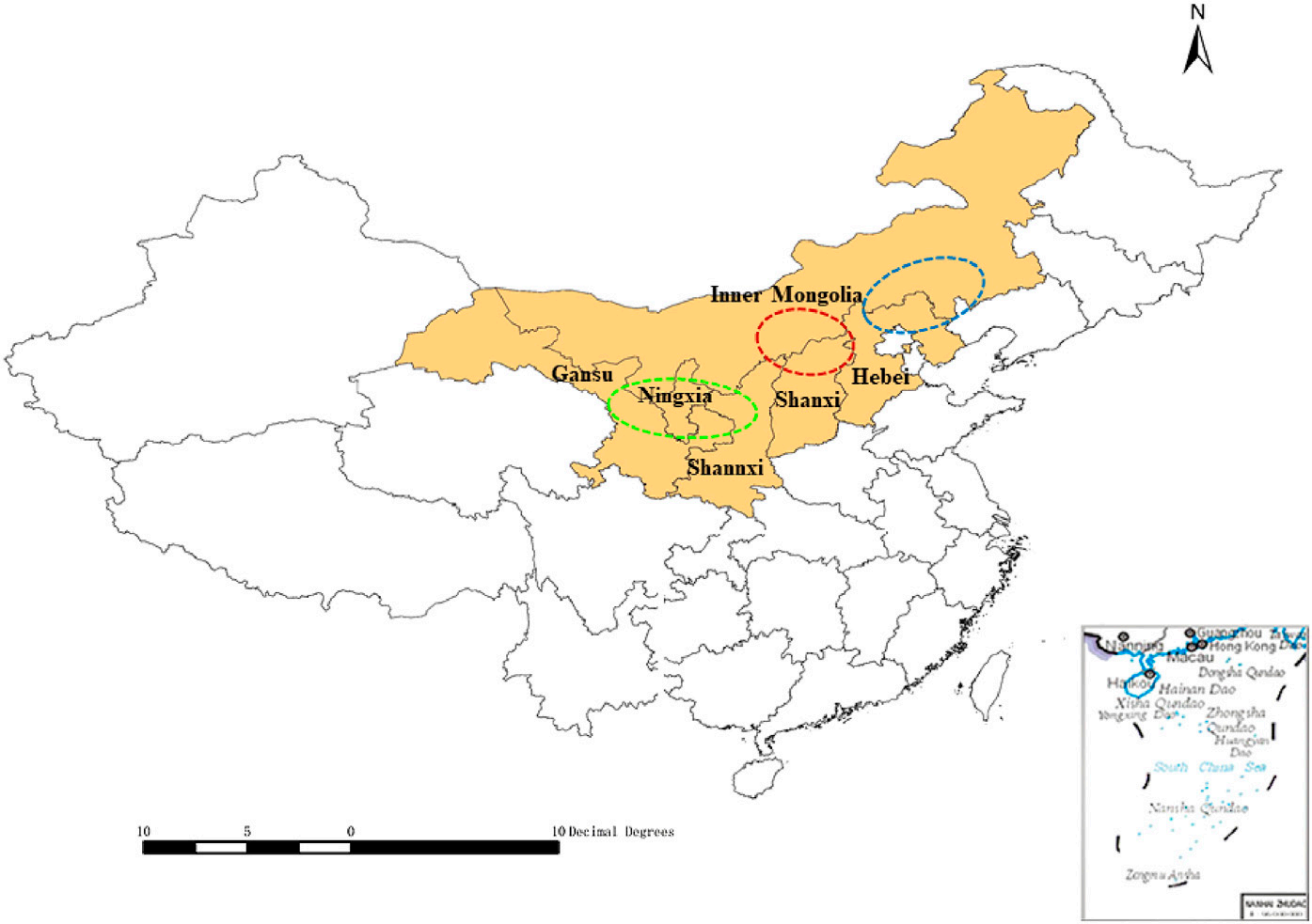
Radix Astragali production areas in China (mapped using ArcGIS v10.4.1). Red circle: center of the *daodi* area of Radix Astragali (Midwest Inner Mongolia and north Shanxi), blue ellipse: main production areas of Radix Astragali in the east (east Inner Mongolia and parts of Hebei), green ellipse: main production areas of Radix Astragali in the west (Midwest Gansu and parts of Ningxia and Shaanxi).

As the demand for medicinal plants in the domestic and international pharmaceutical markets has increased, artificial cultivation has had a positive effect on the protection of wild resources and has allowed market demands to be met (Kling, 2016; Cunningham et al., 2018). Since 1950, cultivated RA products have gradually replaced wild materials. The cultivated *A. membranaceus* var. *mongholicus* is a major source of RA, which accounts for an 80% share of the market (Sinclair, 1998; Qin et al., 2013). Based on their particular natural conditions, ecological environments, cultivation technology, and postharvest processing methods involved (Huang et al., 2011), the Midwest area of Inner Mongolia and north Shanxi Province have been considered as “*daodi*” areas for RA (an area producing top quality herbal medicine). While the demand for RA is increasing, its cultivation has expanded from *daodi* areas to many other production areas, such as Gansu, Shaanxi, Ningxia, and Hebei Provinces (Zhang et al., 2019). The cultivation area and *daodi* region of RA are illustrated in Figure 1.

The cultivation of medicinal plants is helpful for the conservation of their wild resources – it can also be of benefit to farmers economically, and provides social benefits (Booker et al., 2016; Kling, 2016); however, certain quality problems arise when unsuitable planting habitats and intensive input of herbicides, fertilizers, and pesticides are used, and such changes have subsequently resulted in the quality and yield of the medicinal material becoming uneven (Booker et al., 2014; Heinrich, 2015; Yao et al., 2018b). While the supply system of medicinal plants is much affected by its VCs (Booker et al., 2012; Yao et al., 2018b), hence, in order to meet the steadily increasing demand for RA, reconsider the quality measurement system and VCs among suppliers currently used needs to be reconsidered.

VCs describe the different activities involved in different production approaches, i.e., starting from a farmer preparing their raw materials, to the sale of the finished product and the customer (Chopra and Meindl, 2004). They also emphasize the relationships between primary producers and other stakeholders in different production systems and their impacts on the social economy. They have been referred to as a ‘centerpiece of agricultural policy’ in the *World Development* Report 2008 (World Bank, 2007) and are considered to be an important measure for achieving poverty reduction in developing countries.

By understanding the relationship between production and supply, we can identify the product flows, financial flows, and information flows involved in different models. It is then possible to get a better understanding of why the quality and price of herbal medicines vary widely in different markets. In VCs, companies can employ both vertical and horizontal integration as they undertake their production collaborations, export, and e-commerce activities. Hence, VCs can have a series of impacts on the quality of the products, resulting in economic benefits, and effects on patients (Booker et al., 2012; Yao et al., 2018a). Presently, the focus is not just on the economic benefits of a VC, but also on achieving a balance with its effect on the ecological environment and product quality.

Published research shows that chemical components were identified in the RA, include saponins, flavonoids, nitrogen containing chemicals, and aminophenols (Zhang et al., 2019). Among the many compounds present in RA, astragaloside and calycosin-7-O-*β*-D-glucoside are considered to be the main active ingredients. They have potent pharmacological activity and are often used as target compounds to assess the quality of the RA (Zhan et al., 2017). They are also used as “marker components” in various standards in the Chinese Pharmacopoeia (CPC, 2015), and astragaloside is recorded in the European Pharmacopoeia and British Pharmacopoeia (HMSO, 1963; European Pharmacopoeia, 1993).

From the perspective of VCs, the study aims to achieve the quality control strategy of RA. The study was mainly carried out in Inner Mongolia, which is the main production and *daodi* area for RA, in terms of economic, medicinal value and traded volume of plants. This work was based on information arising from an investigation conducted in the main areas of RA production, including Gansu, Shanxi, Shaanxi, Hebei, etc. Differences of the stakeholders, financial flows, and information about RA from production to consumer among its VCs were elucidated. The quality of RA was measured based on its main active ingredients (Cao et al., 2019b). We further described the effects of geographical indication on the quality and yield of the medicinal material and the competitive advantage of vertical coordination. Hopefully, the study will be helpful for the sustainable supplement of high-quality RA.

## Materials and Methods

### Reagents and Chemicals

The acetonitrile used in the high-performance liquid chromatography (HPLC) system was purchased from Thermo Fisher (Thermo Fisher Scientific, United States). Purified water was obtained using Water Purification Systems (Shenyang, China). Formic acid was purchased from the first chemical company of Nanjing (Jiangsu, China); all other reagents were of analytical grade. The reference compounds of astragaloside and calycosin-7-O-*β*-D-glucoside were purchased from the Pufei De Biotech Company (Chengdu, China). All solvents and samples were filtered through a 0.45-µm filter before injection into the HPLC.

The reagents used for analysis of heavy metal pollutants were HNO_3_ and HClO_4_ both at suprapure-grade. Other reagents were of analytical reagent grade unless otherwise stated. The element standard solutions used for assay of the contents of Pb, Cd, As, Hg, and Cu were supplied by National Institute of Metrology (Beijing, China).

All the analytical standards of the studied pesticides were of high purity and certified upon purchase from the Agro-Environmental Protection Institute, Ministry of Agriculture and Rural Affairs (Beijing, China), as being at a purity of greater than 99%. The solvents, acetonitrile (ACN), acetone, methanol, and n-hexane, were of HPLC-grade (Thermo Fisher Scientific, United States). Magnesium sulfate (MgSO_4_) and sodium chloride (NaCl) obtained from Aladdin (China) were used, with a purity exceeding 99%. Other reagents were of analytical reagent grade unless otherwise stated.

### Plant Material

Thirty-one batches of RA samples were collected from 16 habitats across Inner Mongolia. A few samples from Shanxi and Gansu Provinces were used as a control. Firstly, samples purchased in medicinal markets were separated, as their information was thought to be confusing and their origins were uncertain. Secondly, the collected samples were numbered using digital information related to the time and serial number of the collection location. Meanwhile, voucher specimens of the plants were collected and deposited at Baotou Medical College, University of Inner Mongolia. Retaining their plant morphological characteristics allowed them to be examined by personnel with extensive expertise in plant knowledge so as to yield the best possible botanical identification of the samples. Finally, we analyzed 31 samples with complete information after identification.

### Fieldwork

The RA harvest stretches from September to November. The fieldwork presented in this work was carried out during the cultivation and harvesting periods in 2018–2019, and involved a variety of locations in China: Guyang County, Wuchuan County, Helin County, Duolun County, Shangdu County, Naiman County, Siziwang County, etc. in the autonomous region of Inner Mongolia, and Min County and Longxi County in Gansu Province. Based on the characteristics of RA in the supply chain, we modified and designed three different structural questionnaires (Yao et al., 2018a). Semi-structured interviews were also taken from different stakeholders in Gansu, Shanxi, Inner Mongolia, etc. (Figure 1). These have been the main areas of RA production, where cultivation of RA needs 2–3 years. Semi-structured interviews and key informant interviews were conducted with experienced participants in the RA industry. In total, 34 farmers, 16 members of agricultural cooperatives or planting companies, three middlemen, 10 managers of processing companies, and 21 retailers provided information on the production, processing, and retail aspects of the RA industry.

The investigation into the average yield and price of RA in local outlets was supported by the Information and Technology Service Center for Modern Chinese Materia Medica Resources Dynamic Monitoring (founded in 2012) and China Agriculture Research System CARS-21 (founded in 2017). Before the investigation, staff using these two platforms conducted extensive investigations on the cultivation and marketing of RA, respectively, providing us with the basic information we required on the stakeholders. In addition, a large number of RA samples were obtained from the *daodi* and other production areas.

### Quantitative Analyses of Astragaloside in Radix Astragali

A total of 31 batches of RA were analyzed by a HPLC system equipped with an evaporative light scattering detector and diode-array detector. Analysis was performed using a Thermo Fisher Ultimate 3000 HPLC system, and an Agilent C18 column (250 mm × 4.6 mm, 5 μm) with a flow rate of 1.0 ml/min (Du et al., 2014; Chen et al., 2015). The mobile phase consisted of acetonitrile and deionized water (32%:68%). The injection volume was 10 μl, and the column component was set to a temperature of 30°C.

### Quantitative Analyses of Calycosin-7-O-*β*-D-Glucoside in Radix Astragali

Analysis was performed using a Waters C18 column (250 mm × 4.6 mm, 5 μm) at a flow rate of 1 ml/min. The mobile phase consisted of acetonitrile (solvent A) and water (containing 0.2% methanoic acid, solvent B). Gradient elution was applied as follows: 0–20 min, 80–60% B; 20–30 min, 60% B. The injection volume was 10 μl, and the temperature of the column was maintained at 30°C. Detection was performed at a wavelength of 260 nm. Each sample was assayed in triplicate.

### Detection of Heavy Metal Contaminants

Due to the possibility of heavy metal contamination, it is necessary to determine the contents of harmful substances, thus to ensure the safety of the products. Amount of Pb, Cd, As, Hg, and Cu in RA were analyzed by atomic absorption spectrometry (AAS) (CPC, 2015). A Thermo ICE 3000 atomic absorption spectrometer with deuterium background corrector was used in this study. Pb and Cd contents in plant samples were determined by HGA graphite furnace using argon as the inert gas. Cu was assayed out in an air-acetylene flame. Other measurements were based on cyanide complex processing (Tüzen, 2003; CPC, 2015).

### Detection of Pesticide Residues

The organochlorine residues including total BHC (α-BHC, β-BHC, γ-BHC, δ-BHC), DDT (pp'-DDE, pp'-DDD, op'-DDT, pp'-DDT), and pentachloronitrobenzene (PCNB) were determined in accordance with the following gas chromatography tandem mass spectrometry method (GC-MS) (CPC, 2015). A Thermo TRACE 1300 gas chromatograph was used. Nitrogen with a purity of 99.99% was used as the carrier gas. A DB-5MS capillary column (0.25 mm × 30 m × 0.25 µm) was used. A full auto-tune of the mass spectrometer was performed before the analysis of each set of samples. The transfer-line temperature was 300°C, the manifold temperature was 50°C, and the ion-trap temperature was 250°C. The flow rate was 1.0 ml/min and the sample injection volume was 1 µl.

### Value Chain Analysis

The analysis of the different VCs was undertaken in four stages: 1) identify the main VCs and add the stakeholders to the corresponding procedures; 2) calculate the market price of each trading link based on the interview data and information from the RA industry and then convert this into CNY/kg; 3) analyze the strengths and weaknesses of the VCs in relation to safety, quality, and geographical indication; 4) draw up a framework for the VCs to relate the main production activities to the stakeholders. Additionally, the production behavior, quality, and financial performance of the RA in VCs were analyzed according to Yao et al. (2018b).

### Clustering Analysis

The geographical indication of a medicinal plant is related to the geographical location where the plant is grown. *Daodi* herbal medicines have adapted to their environments over long periods of time and have self-adapting characteristics. They are thus thought to feature superior qualities, including superior active ingredients and yields.

The *k*-means algorithm is a clustering method based on partitioning which is capable of effectively dealing with large-scale data but is easy to understand (Diao, 2012). Contents of the two measured constructs and yields of the fresh roots were combined into a data frame. The R-tree indexing algorithm was then used to analyze the spatial index in the *k*-means clustering method.

## Results and Discussion

### Industrial Structure and Value Chains

RA cultivation began in the 1950s and was intended to mitigate the sharp decline in wild populations while meeting the demands of commercial enriches. Later on, RA was artificially cultivated in Gansu, other parts of Inner Mongolia, and Shanxi on a large scale. *A. membranaceus* var. *mongholicus* is the main source of RA, and is cultivated much more than *A. membranaceus*, providing 80% of the RA medicinal market. Therefore, the present study launched a series of investigations into the production of *A. membranaceus* var. *mongholicus*.

**FIGURE 2:**
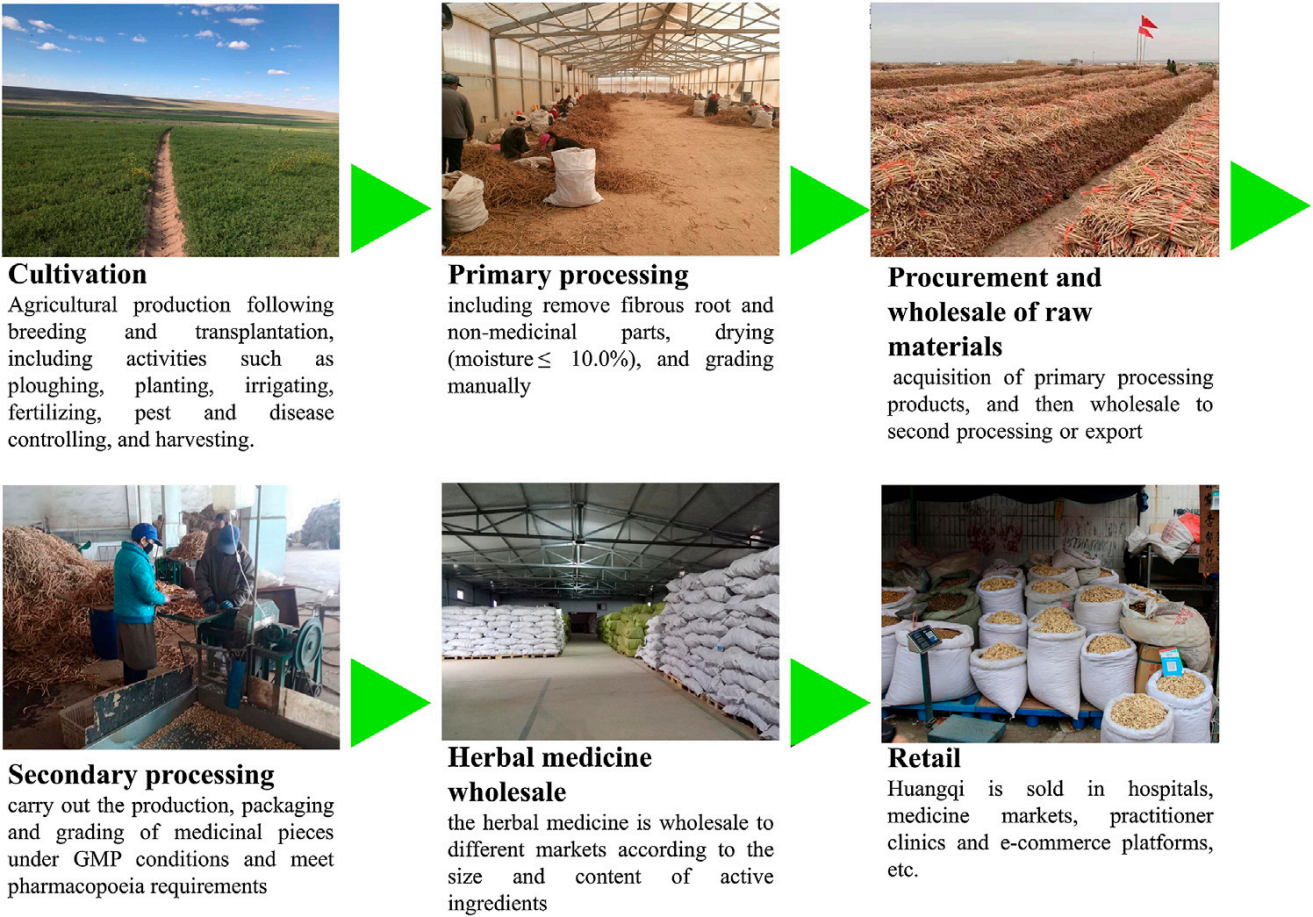
Radix Astragali goes through six stages before it reaches the consumer.

Inner Mongolia has long been considered as a *daodi* area of RA, with high production rates and superior quality. As such, RA was popularly used in traditional Chinese medicinal practice. With its long production and supply history, the practice of RA production is found to consist of 10 mature VCs, which can be distinguished by their various composite patterns of stakeholders. Typically, RA goes through six production stages until it reaches the herbal wholesale and retail markets shown in Figure 2.

**FIGURE 3:**
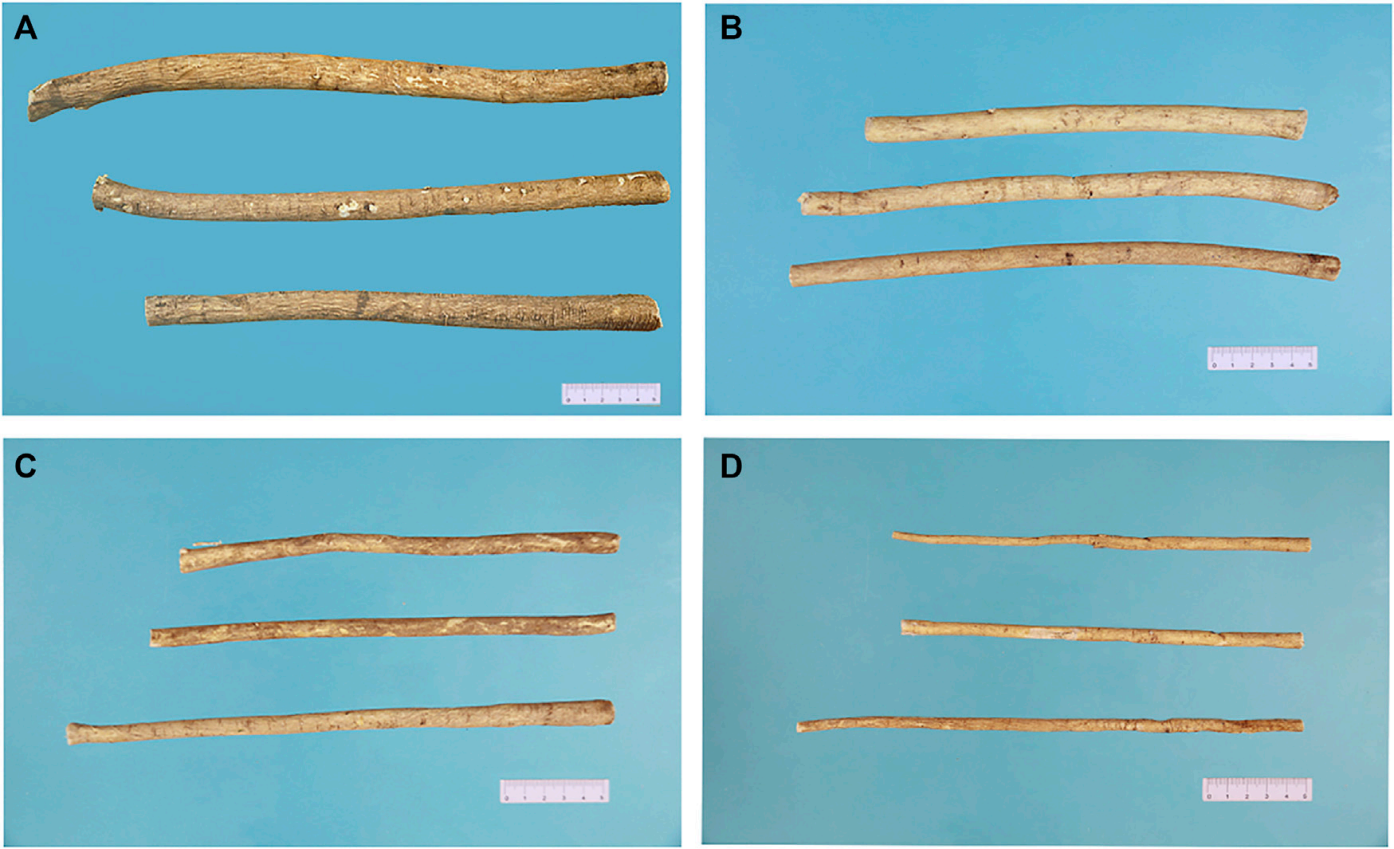
Commercial grades of Radix Astragali found at a local market in Inner Mongolia: **(A)** huge (d > 1.5 cm), **(B)** large (*d* > 1.2 cm), **(C)** medium (1.2 cm ≥ *d* > 0.8 cm), and **(D)** small (0.8 cm ≥ *d* ≥ 0.35 cm).

Although RA is available in a diverse range of forms, dried roots and traditional Chinese decoction pieces are still the main consumables. The price varies with quality, and is consistent with the size and active ingredients. The Chinese Pharmacopoeia (CPC, 2015) gives the standards for the quality control of RA. In addition to public standards, there are also a number of different private market standards and these can be broadly divided into four grades. Commonly, based on size, different prices are often found for RA that is “small” (0.35–0.8 cm), “medium” (0.8–1.2 cm), “large” (>1.2 cm), and “huge” (>1.5 cm), as illustrated in Figure 3.

**FIGURE 4:**
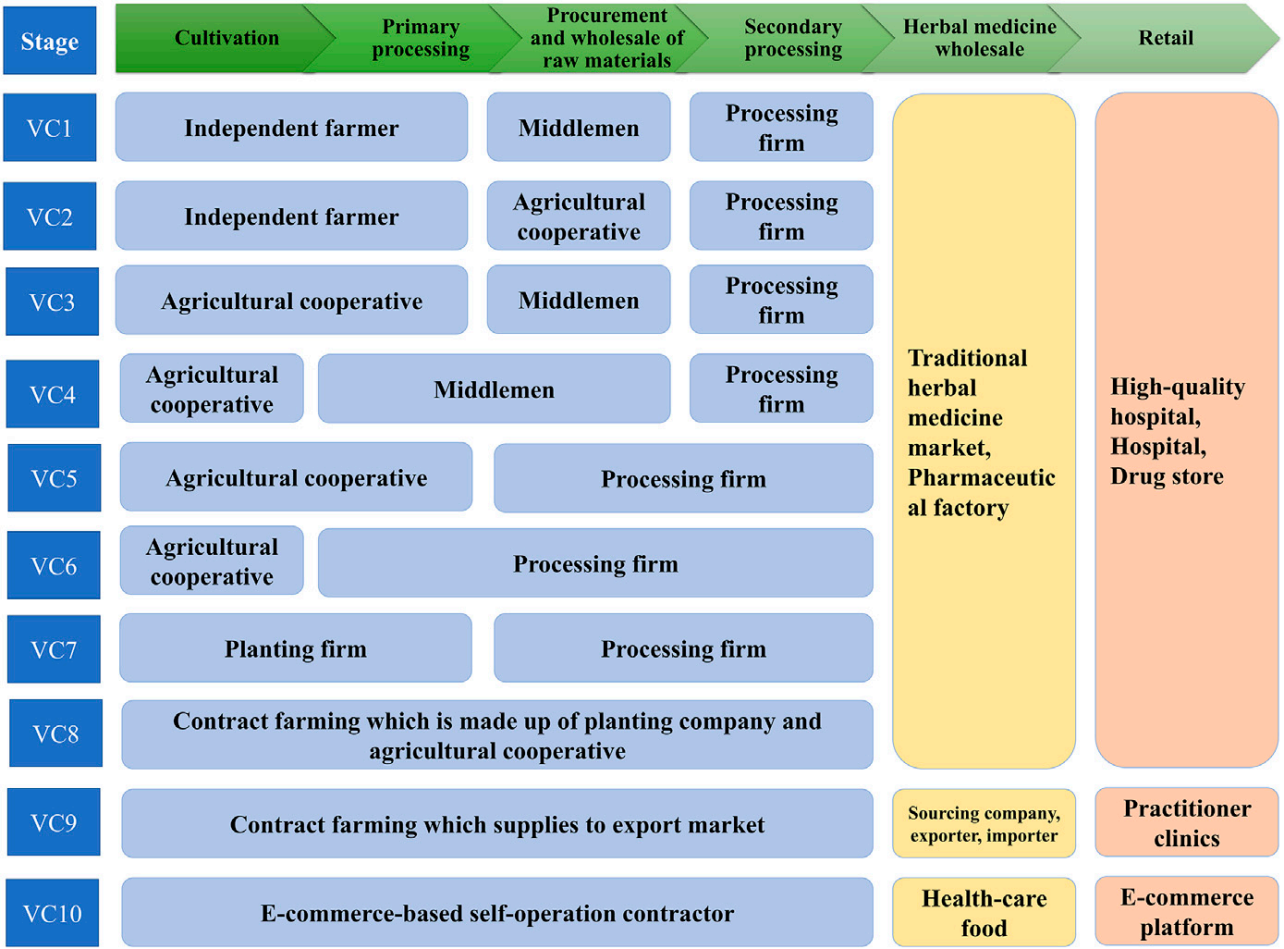
Primary value chains and stakeholders involved in Radix Astragali produce.

Figure 4 demonstrates the 10 primary VCs identified for RA and the stakeholders involved. Different stakeholders may have various roles, and the division of labor is also slightly different. Among the 10 VCs, middlemen or agricultural cooperatives play intermediary roles in VCs 1–4, while in VCs 5–10 production is made to order which weakens the role of middlemen.

VCs 1 and 2 begin with independent farmers with relatively small RA fields (<2 ha). These tend to be of traditional, small-scale, peasant economy form, and have been an important part of RA production for decades. Most of the time, the RA is delicately processed and graded in order to gain more profit, while the exception is when cash is scarce and they are sold as fresh roots. In these VCs, farmers often sell RA through large suppliers, such as middlemen, cooperatives, etc., and these procedures lead to high transaction costs and switching cost. Although the yields of RA products are sometimes higher than in other stages, procurement difficulties limit farmers’ incomes. Farmers near traditional herbal medicine markets or processing companies can sell their products directly to increase profits earned from cultivation of this medicinal plant. The model is especially prevalent in the parts of Gansu and eastern Inner Mongolia where RA is cultivated.

VCs 3–6 begin with agricultural cooperatives that have relatively large fields (∼30 ha). These are gatherings of several farmers who took over large fields and invested in more machinery than independent farmers. Cooperatives purchase equipment and facilities to cultivate and process for the common good. Farmers involved in this stage would produce RA material individually or cooperatively and make it wholesale. The farmers can easily benefit from financial and technical supports from local government, and gain financial resources in the form of bank credits. In addition, after a few years, many cooperatives that participate in RA planting become inclined to sell their fresh roots to middlemen or processing companies, thereby achieving rapid capital gains for the following year’s planting.

VC 7 begins with planting companies. These are large plantations (>60 ha) with high degrees of mechanization, comprehensive sprinkler–irrigation systems, and storage warehouses. The planting companies buy seeds from large breeders or specialized seed companies and hire farm workers to do the farm work. This model is especially prevalent in Midwest Inner Mongolia, as much land is available for RA cultivation therein.

VC 8 is based on contract farming which involves vertical coordination within the chain. The planting company is involved in all the nodes of the VC, from production, through processing, to wholesale. This involves the formation of verbal or written agreements by the planting company, which bridges the gaps between farmers and trading companies or other production enterprises. Owing to fewer intermediate links existing between suppliers with retailers, the costs have been reduced and the economic efficiency is improved. All stages are all traceable, including the production chains and planting techniques, the application of fertilizers and pesticides, and quality inspection. In this model, the company can provide farmers with production targets and standards before the annual production process begins, as well as fixing the current purchase price. However, there are signs that farmers are starting to form alliances with local planting or processing companies (thus to change the traditional route of selling products locally) for the purpose of getting better quality products and entering more profitable markets. Studies have also shown that some procurement agencies (planting or processing companies) prefer to work with large-scale farmers rather than smallholder farmers because of the higher transaction costs associated with dealing with the latter. Commercial markets are highly competitive, with high quality standards and requirements of consistent, timely deliveries, which smallholders struggle to achieve (Reardon et al., 2009; Wainaina et al., 2012; Da Silva and Rankin, 2013; Namulindwa, 2018).

VC 9 involves another form of integrated vertical coordination in which products are directly supplied to export markets. In VC 9, the botanical raw materials begin with medicinal material growers in China, which are then exported to consumers in foreign countries via the export markets. In this VC, some of the RA is used for medicine for treatment or health care under the guidance of traditional practitioners, and some is finely processed by the drug manufacturer. Furthermore, “added value” occurs due to the processing which is carried out by the farmers, processers, export and import middlemen, suppliers, and foreign herbal practitioners. The planting company becomes linked with the sourcing companies (i.e., joint ventures or big pharma) in order to enter the export market. In the process, the stakeholders need to pay careful attention to the quality of the products in order to improve the reliability of the RA production system and achieve reputational and marketing goals. RA agriculture also encourages producers to implement the Hazard Analysis and Critical Control Point System (HACCP). However, the RA produced in this VC is exported through a complicated series of quality inspection and intermediate sales procedures, without second processing, and is finally sold in the export market as prescription drugs or health food.

VC 10 is a relatively new model based on e-commerce. Although the sales information accompanying the goods often states that the RA was “collected in the wild,” in fact this is not necessarily true. In the process of VC, the contractor (in the form of a middleman or small-scale medical processing plant) is the main participant. They procure RA with good appearance, process it into pieces or powder, and package it more attractively before selling it on the network platform. In theory, e-commerce should provide higher profit than conventional markets as operating costs are highly reduced; however, the quality control of such products sold via the network platform needs to be greatly improved.

### Financial Performance of Stakeholders

**FIGURE 5:**
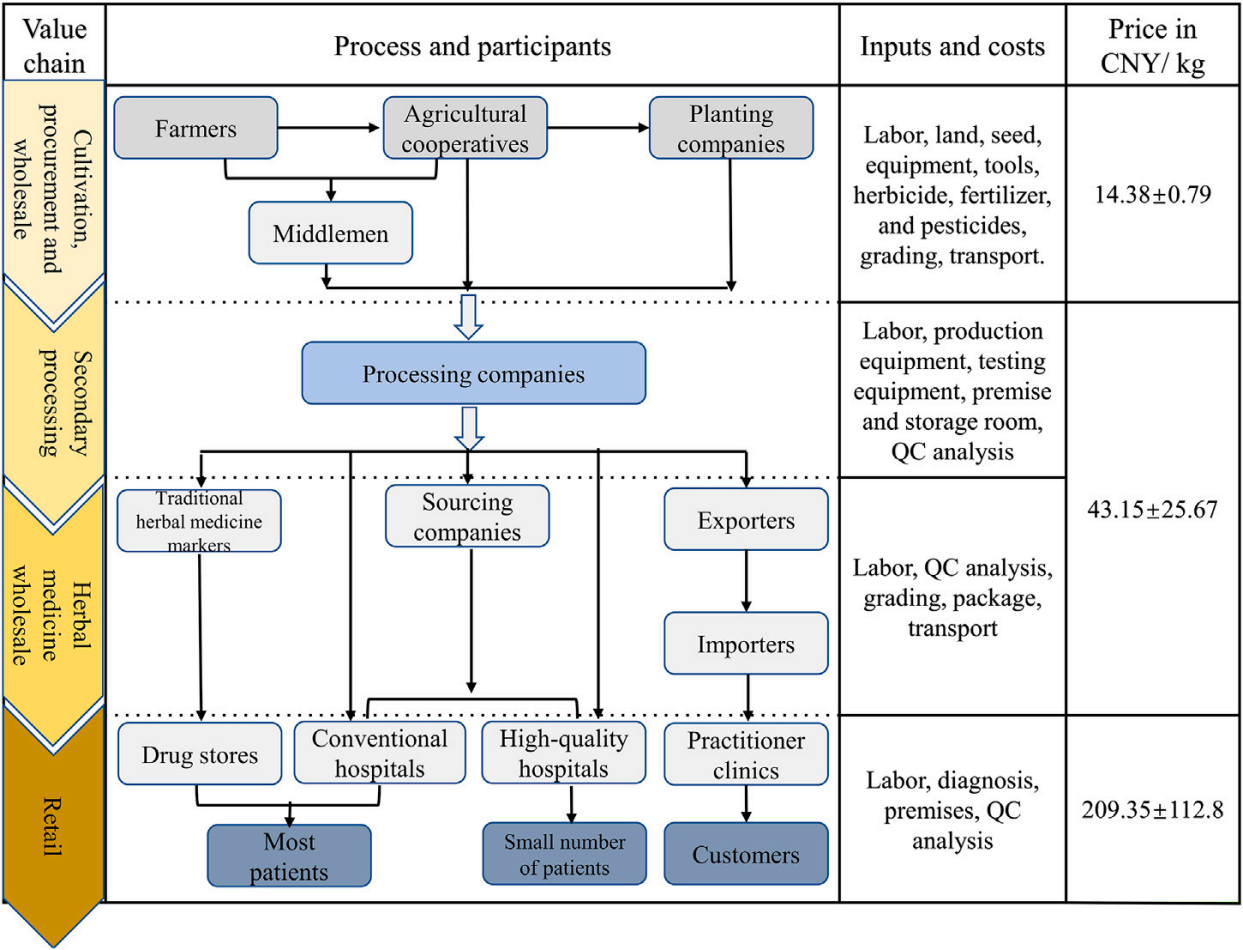
Herbal practitioner value chains for crude botanical materials, beginning with a grower in Inner Mongolia and finishing with the consumers. The data were derived from informants questioned after the Radix Astragali harvest in 2019. Value are expressed as Mean ± Srandard deviation (SD).

The monetary value of herbal medicine is created by the stakeholders’ production activities and is generated during the trading process. Labor and non-labor inputs promote the value of the products to different extents in different VCs. Stakeholders also play different roles at the same stage, so they can take different risks and accrue various benefits in the process (Yao et al., 2018b). Figure 5 illustrates the value variability of RA products along its way from farmer to consumer.

At the cultivation stage, stakeholders are attracted to RA agriculture due to the greater monetary value attached to medicinal plants (12,000–22,500 CNY/ha) compared to crop production (7,500–12,000 CNY/ha). However, prices in the medicinal-materials market are subject to intense fluctuations. The difference has a strong influence on the acreage devoted to such raw materials (both positive and negative). All of the costs are relatively high during the cultivation stage. Non-labor costs at this stage consist of land, seed, equipment, tools, irrigation pipes, herbicides, fertilizers, and pesticides, etc. There is an enormous demand for labor as the seeding, weeding, and harvesting machinery is semi-automatic, especially for the green production practices. Labor is required to care for the field management, transplant, harvest, dry, process, and transport the materials, and required for manual grading, etc. Collectively, local farmers need to pay 130–200 CNY to each person per day for such labor services.

In addition, some stakeholders (farmers, agricultural cooperatives, and planting companies) process the plants themselves, except when there is large economic pressure whereupon they will sell the fresh roots at low prices. Among the various models, the independent farmers in VCs 1 and 2 will carry out breeding by themselves and use their own farmland for cultivation purposes. This will help them to reduce non-labor costs, so as to save input while ensuring higher returns. Additionally, during the harvesting process, the drying and primary processing processes can all be performed by family members in order to save labor costs. In contrast, the large-scale agricultural operations involved in VCs 3–9, which are becoming more popular, including agricultural cooperatives and planting companies, pay more attention to their economic benefits and production efficiency.

Procurement and wholesale of raw materials are key processes in the VCs. Some VCs use middlemen in these processes but sometimes the functions of such middlemen are omitted or replaced by agricultural cooperatives. At this point, suppliers that are closer to the processing companies and get more information about transactions are more likely to make direct contact with the contractor, without the need for involving middlemen. Procurement costs include procuring and reselling labor, traveling costs, and transportation costs. Middlemen need to invest a part of their original capital in the procurement process, so financial business credit is very important to them. Also, as the product quality of the RA from different sources varies, and considering the ease with which information can be communicated, the pricing structure of the RA is affected by the locality of the cultivation area.

The second processing and wholesale stages are also labor-intensive and include washing, cutting into pieces, re-drying, hand selecting, size-based grading, and transportation. Only VC 9 does not require the RA to be cut into. Before they carry out the second processing stage, processing companies have to spend a lot of money on production and testing equipment, production workshops, and storage rooms. In addition, their factories need to perform quality testing (according to the Chinese Pharmacopoeia) for each batch of traditional Chinese medicine processed. This is also where a large part of the input is made in the management of the factory.

The middle stage of the transformation process involves the wholesale of the herbal medicine. The stakeholders are engaged by the traditional herbal medicine market, pharmaceutical factory, sourcing company, and contractor. After processing, the herbal medicine is sold wholesale to different markets according to the sizes and contents of the active ingredients. The aim is to supply high-quality RA to their customers at a favorable price which fluctuates between 20 and 100 CNY/kg.

Retail is the last stage in the RA value chain shown in Figure 5. Here, the resources flow to high-quality hospitals, traditional herbal medicine markets, export markets, and online markets. The price of the RA now ranges from 50 to 330 CNY/kg. Differentiated commodity values are reflected in the different markets, so that the selling price to the export market and high-quality hospitals is more than three times that to other retailers. Studies of the trends in prices show that they are related to the dynamic balance between demand and supply. It is thus thought that as the demand for RA products grows, progressively more and more of the RA will be harvested. Then, the prices (and farmers’ financial gains) will decline.

### Geographical Indication

*Daodi* herbs are famous in traditional Chinese medicine. They have a high and consistent quality which, in addition to their place of origin and its ecological conditions, should be defined by their traditional methods of culture and production, and even their specific germplasm (Booker et al., 2012).

During our study, samples were analyzed to see if there are any differences between the RA from *daodi* and other areas. Quality of RA from different origins and quality standards are commonly adjudged by the index composition of astragaloside or calycosin-7-glucoside (Cao et al., 2019a; Cao et al., 2019b; Xue et al., 2019; Tian et al., 2020). Liu et al. (2019) shows that compared with flavonoids and other isoflavones, calycosin-7- glucoside makes a significant contribution to distinguishing RA from different origins. Therefore, we analyzed 31 batches of RA and classified them using a classification scheme based on three variables including the yield of medicinal material, and the contents of astragaloside and calycosin-7-glucoside (Supplementary Material show the chromatography).

**FIGURE 6:**
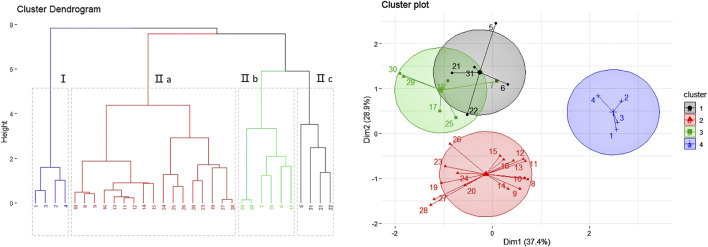
Results of the geographical indication analysis (based on 31 batches of Radix Astragali from 16 habitats).

Table 1 displays the results of the content analyses. Clustering analysis (using the *k*-means algorithm and R v3.6.1 software package) was then adopted to compare the samples. The results are illustrated in Figure 6 in the form of a dendrogram. In Figure 6, the samples collected from Inner Mongolia, Shanxi and Gansu Province are divided into different clusters (labeled Ⅰ and Ⅱ). Cluster Ⅱ is further divided into three subgroups (labeled a, b and c) based on geographic variation. The contents of active ingredients in the RA from Gansu Province are found to be lower than that in the products from Inner Mongolia; however, the yield of medicinal material is higher. The RA *daodi* herbal medicines from specific areas of Guyang, Helin, Wuchuan, and Wulateqian counties are of better quality than that from other habitats due to their natural geographical environments and human factors, etc. The results of our geographical indication evaluation were universally accepted by the stakeholders involved in the production and supply chains. The RA also includes other saponins, flavonoids, polysaccharides, amino acids, etc. Researchers have tried to establish comprehensive quality evaluation protocols for RA by using fingerprinting methods (Zhang et al., 2016). Future research will we need to explore the comprehensive influence of multi-index components on quality control indices and evaluate resources for RA.

**TABLE 1:** The results of the content analyses performed on the 31 batches of Radix Astragali samples.

Sample no	Cluster	Longitude	Latitude	Calycosin-7-O-*β*-D-glucoside (%)	Astragaloside (%)	Yield (kg/mu)
2019001GS	Ⅰ	E104°14′54.27″	N35°0′15.28″	0.034	0.032	750
2019002GS	Ⅰ	E104°02′3.19″	N34°26′50.11″	0.041	0.076	800
2019003GS	Ⅰ	E104°35′35.64″	N34°59′55.64″	0.034	0.042	775
2019004GS	Ⅰ	E103°58′9.44″	N34°21′13.7″	0.032	0.063	750
2019005IM	Ⅱ c	E113°11′29.69″	N41°23′49.50″	0.049	0.159	600
2019006IM	Ⅱ b	E111°14′12.04″	N41°33′22.08″	0.043	0.087	590
2019007IM	Ⅱ b	E116°34′08.86″	N42°20′57.02″	0.024	0.053	540
2019008IM	Ⅱ a	E110°15′01.69″	N41°21′10.24″	0.064	0.035	640
2019009IM	Ⅱ a	E110°35′12.10″	N40°50′09.45″	0.064	0.025	625
2019010IM	Ⅱ a	E109°56′03.12″	N40°37′40.66″	0.058	0.026	640
2019011IM	Ⅱ a	E111°47′41.95″	N40°20′14.27″	0.061	0.050	665
2019012IM	Ⅱ a	E111°51′17.16″	N40°24′14.43″	0.058	0.047	665
2019013IM	Ⅱ a	E111°39′51.05″	N40°16′21.62″	0.060	0.050	655
2019014IM	Ⅱ a	E111°45′22.29″	N40°06′22.07″	0.070	0.040	655
2019015IM	Ⅱ a	E112°0′31.96″	N40°06′17.19″	0.065	0.065	655
2019016IM	Ⅱ a	E112°10′24.61″	N40°27′9.07″	0.057	0.047	655
2019017IM	Ⅱ b	E120°48′10.48″	N42°21′06.52″	0.046	0.067	525
2019018IM	Ⅱ b	E113°51′55.26″	N41°34′03.00″	0.023	0.055	525
2019019IM	Ⅱ a	E111°38′12.17″	N41°37′50.87″	0.069	0.041	550
2019020IM	Ⅱ a	E110°39′24.78″	N40°24′43.84″	0.077	0.057	625
2019021IM	Ⅱ c	E110°44′07.09″	N40°42′14.23″	0.057	0.133	630
2019022IM	Ⅱ c	E111°11′14.06″	N40°16′22.04″	0.058	0.094	650
2019023IM	Ⅱ a	E109°36′12.46″	N40°56′42.16″	0.081	0.084	625
2019024IM	Ⅱ a	E109°49′51.91″	N40°53′02.79″	0.056	0.031	610
2019025IM	Ⅱ a	E109°00′05.35″	N40°51′21.68″	0.042	0.061	620
2019026IM	Ⅱ a	E111°20′16.89″	N41°12′4.38″	0.055	0.06	625
2019027IM	Ⅱ a	E111°35′58.07″	N41°04′46.71″	0.078	0.046	610
2019028IM	Ⅱ a	E110°54′35.45″	N40°59′49.19″	0.079	0.044	610
2019029IM	Ⅱ b	E115°47′40.67″	N42°52′49.60″	0.023	0.064	525
2019030IM	Ⅱ b	E115°48′42.67″	N42°52′09.90″	0.022	0.066	525
2019031SX	Ⅱ c	E113°40′0.88″	N39°42′54.81″	0.032	0.102	725

IM, Inner Mongolia; GS, Gansu; SX, Shanxi.

### Radix Astragali Quality in the Different VCs

Various voluntary and mandatory standards, regulations, and requirements are used in the production and retailing of herbal medicines to control the quality and safety of the final products. Measures that can be used to evaluate the quality of RA have been established in pharmacopoeia. Characterization tends to be made according to the appearance, transection, nature, and flavor of RA, and on certain tests, including chemical identification, quantification analysis, and residual amounts of pesticides and heavy metals (CPC, 2015).

In high-quality RA, the roots have cuticles that are smooth and pliable. Their cross-sections are dense and their textures are solid, and the center of the xylem is light yellowcolor. The product quality and safety of the plants can be accurately determined using a combination of traditional and modern identification and analytical quantification techniques. As a result, the supply of these globalized commodities is improved considerably.

The contents of total Pd, Cd, As, Hg, and Cu vary between 0 and 2.4061 mg/kg, 0 and 0.0369 mg/kg, 0.0112 and 0.2213 mg/kg, 0 and 0.1125 mg/kg, 4.7928 and 9.2858 mg/kg, with an average concentration of 0.6165, 0.0054, 0.0839, 0.0362, and 7.0418 mg/kg, respectively. The contents of heavy metals in RA comply with the limit standard of Chinese Pharmacopoeia, as well as the standards of the natural traditional Chinese Medicine materials, and standards of China Hong Kong, Japan, China Taiwan, United Kingdom and the United States (BS, 2015). From the measured pesticide residues, it was observed that the residues of BHC, DDT, and PCNB were found in samples at levels below 0.0488, 0.0023, and 0.0015 mg/kg, which were below permissible limits. The concentration of organochlorine pesticide residue was found to be within safe limits. Heavy metal and pesticide residues were found to be up to the standard as found in the study (Table 2).

**TABLE 2:** The contents of the heavy metal and pesticide residues on the 31 batches of Radix Astragali samples.

Sample no	Heavy metal (mg/kg)					Pesticide residues (mg/kg)		
	Pb	Cd	As	Hg	Cu	BHC	DDT	PCNB
2019001GS	0.6571	—	0.0416	0.1018	6.3993	—	—	—
2019002GS	0.1265	—	0.0335	0.0078	7.6253	0.0418	—	—
2019003GS	0.1466	0.0113	0.0640	0.0012	6.9752	0.0409	0.0023	—
2019004GS	0.5724	0.0001	0.0501	0.0312	7.1044	0.0238	0.0023	—
2019005IM	0.0811	0.0052	0.0956	—	7.8815	0.0095	—	—
2019006IM	0.1914	0.0095	0.0255	0.0572	9.2858	0.0488	0.0012	0.0010
2019007IM	0.0739	—	0.0112	—	6.3453	—	—	—
2019008IM	0.0609	—	0.1447	0.0156	8.6778	0.0091	—	—
2019009IM	2.4061	0.0369	0.1631	0.0638	8.7489	—	—	0.0012
2019010IM	0.3471	0	0.0960	0.1125	8.5240	0.0057	—	—
2019011IM	—	—	0.2023	0.0124	4.7928	–	–	—
2019012IM	1.1124	0.0065	0.2213	0.0061	6.5630	0.0061	0.0010	—
2019013IM	0.7809	—	0.0561	—	8.5231	0.0067	—	—
2019014IM	0.5661	0.0091	0.0984	0.0095	5.3323	0.0417	—	—
2019015IM	0.4678	0.0006	0.0675	0.0022	6.3645	0.0074	0.0012	0.0010
2019016IM	0.0923	0.0012	0.0649	—	4.8126	—	—	—
2019017IM	0.9518	0.0057	0.1654	0.0057	5.5663	0.0069	—	—
2019018IM	0.6841	0.0035	0.0129	0.0067	6.0265	0.0218	0.0015	—
2019019IM	0.8594	—	0.0613	0.0912	6.3138	0.0069	—	—
2019020IM	1.6125	0.0073	0.1170	0.0138	8.9820	0.0073	—	—
2019021IM	0.3682	0.0005	0.1280	0.0061	8.0072	—	—	—
2019022IM	0.1215	0.0015	0.0352	0.0483	7.1362	0.0135	0.0023	—
2019023IM	0.0793	0.0047	0.0951	0.0764	7.6247	0.0094	—	—
2019024IM	0.5226	0.0068	0.0642	0.0576	4.8623	0.0063	0.0023	0.0010
2019025IM	0.3601	0.0026	0.1690	0.0915	4.9561	0.0077	—	—
2019026IM	0.9120	0.0034	0.0662	—	8.8135	—	—	—
2019027IM	—	0.0018	0.0268	0.0017	7.8915	—	—	—
2019028IM	0.8941	0.0009	0.0544	0.0152	7.1783	0.0161	0.0017	0.0015
2019029IM	0.6354	0.0003	0.0934	0.0369	6.7128	0.0065	—	—
2019030IM	1.5079	—	0.0142	0.0658	7.6173	0.0092	—	—
2019031SX	0.6892	0.0048	0.0636	0.0052	6.654	0.0069	0.0012	—

It has been shown that there is a strong relationship between good manufacturing practice (GMP) and the traceability of the quality of the RA (Qi et al., 2015). Although the risk-assessment standards used in GMP certification were canceled in 2019, they remain one of the most important tools ever used to guarantee the quality of the manufacturing process. Instead of having to get GMP certification every year, processing companies now have to ready themselves for standard inspections. Quality control measures have been raised during these inspections, and the finished products are guaranteed to be of acceptable quality before they are released for sale (Govindaraghavan, 2008; He et al., 2015).

In more recent times, voluntary standards have also been further developed. These include standards relating to: excellent germplasm [Chinese medicinal plant seeds and seedlings—Seeds of Menggu Radix Astragali (*Astragalus membranaceus* (Fisch.) Bge. var. *mongholicus* (Bge.) Hsiao, 2017]; the distribution of *daodi* areas (*Daodi* herbs—Beiqi, 2018); breeding and cultivation standards [The Seedling transplanting technical specification of *Astragalus membranaceus* (Fisch.) Bge. var. *mongholicus* (Bge.) in Midwest Inner Mongolia, 2018]; the classification of medicinal materials (Commercial grades for Chinese Materia Medica – ASTRAGALI RADIX, 2018), etc.

In addition to published standards, some planting companies and pharmaceutical factories also impose their own regulations to ensure the quality of their herbal medicines. There are many standards that can be used for quality control, but independent farmers are not proactive enough technologically to access these. In contrast, farmers involved in vertically-integrated VCs can get technological advice and support from various companies in the VCs.

In order to meet consumer demand for RA, core stakeholders have attempted to establish traceability frameworks that cover the whole of the supply chain (including planting companies, processing companies, and various markets) based on their domestic and international experience with traceability systems constructed for agricultural and food products. However, a major impediment to advancing the safety of raw RA is the failure of producers to consider their products as agricultural commodities rather than traditional Chinese medicines.

As VCs 1–6 involve multiple sources and intermediary nodes, the information that needs to be collected is more widely dispersed which makes it much more difficult to check the integrity of the traceability chain. VCs 7–8 incorporate traceable management information platforms that record a certain amount of electronic data (covering breeding, cultivation, production processing, and circulation) for the whole supply chain according to specified requirements. Unfortunately, almost all the current traceability technologies in place only provide production or sales information. That is, it excludes information about habitat (precautions taken before planting and details of the natural and geographical environments) and input of agricultural materials (e.g., fertilizers, pesticides, and herbicides). Only the products in VC 9 that link directly to export markets are developed under complete product-to-market control.

In VCs that are not fully vertically integrated, consumers and markets cannot be told the true year and area of production of the RA as this information is unknown due to the incomplete nature of their traceability systems. In addition, the assessment of RA quality will be subject to different hazard risks in different VCs (Table 3). The use of vertically-integrated VCs in medicine production therefore shows some differences compared to the general markets.

**TABLE 3:** Quality of the Radix Astragali for the different value chains and likelihood of risks being made to its quality during its production.

VC	Traceability	Certify	Control	Heavy metal	Pesticide residue	Likelihood of hazard occurring		
						Cultivation	Processing	Procurement
1	No	No	Weak	Seldom	Seldom	Probable	Improbable	Very probable
2	No	No	Weak	Seldom	Seldom	Probable	Improbable	Very probable
3	No	No	Medium	Seldom	Seldom	Probable	Improbable	Very probable
4	No	No	Medium	Seldom	Seldom	Probable	Improbable	Very probable
5	No	No	Medium	Seldom	Seldom	Probable	Improbable	Probable
6	No	No	Medium	Seldom	Seldom	Probable	Improbable	Probable
7	Maybe	Maybe	Strong	Rare	Rare	Improbable	Improbable	Probable
8	Maybe	Maybe	Strong	Rare	Rare	Improbable	Improbable	Probable
9	Yes	Maybe	Strong	Rare	Rare	Improbable	Improbable	Improbable
10	Maybe	Maybe	Strong	Rare	Rare	Improbable	Improbable	Probable

### Relationship Between Behavior, Revenue, and Quality

RA is a traditional herbal medicine and is included in a broad range of products including foods, beverages, cigarettes, toiletries, etc. As demand for RA has grown, the standards used to specify its purity and identification have not always kept pace with the expansion process. As a result, there has been a decline in its quality in the supply chains. Production processes driven by economic value can result in the products having poor quality and suffering from adulteration. In the current context, this means the RA products contain an insufficient amount of the active ingredients, an excessive amount of pesticide residues and/or heavy metals or be mixed with *A. membranaceus* in the seed stage. Herbal medicines with high concentrations of active ingredients are more likely to be purchased by pharmaceutical companies. They are also more likely to be used in adulteration schemes. The problem is more likely to occur in the traditional herbal medicine market, but the form of adulteration only works in the short term. In the longer term, it will seriously affect the credibility of such unscrupulous sellers and the prices they are able to charge.

**FIGURE 7:**
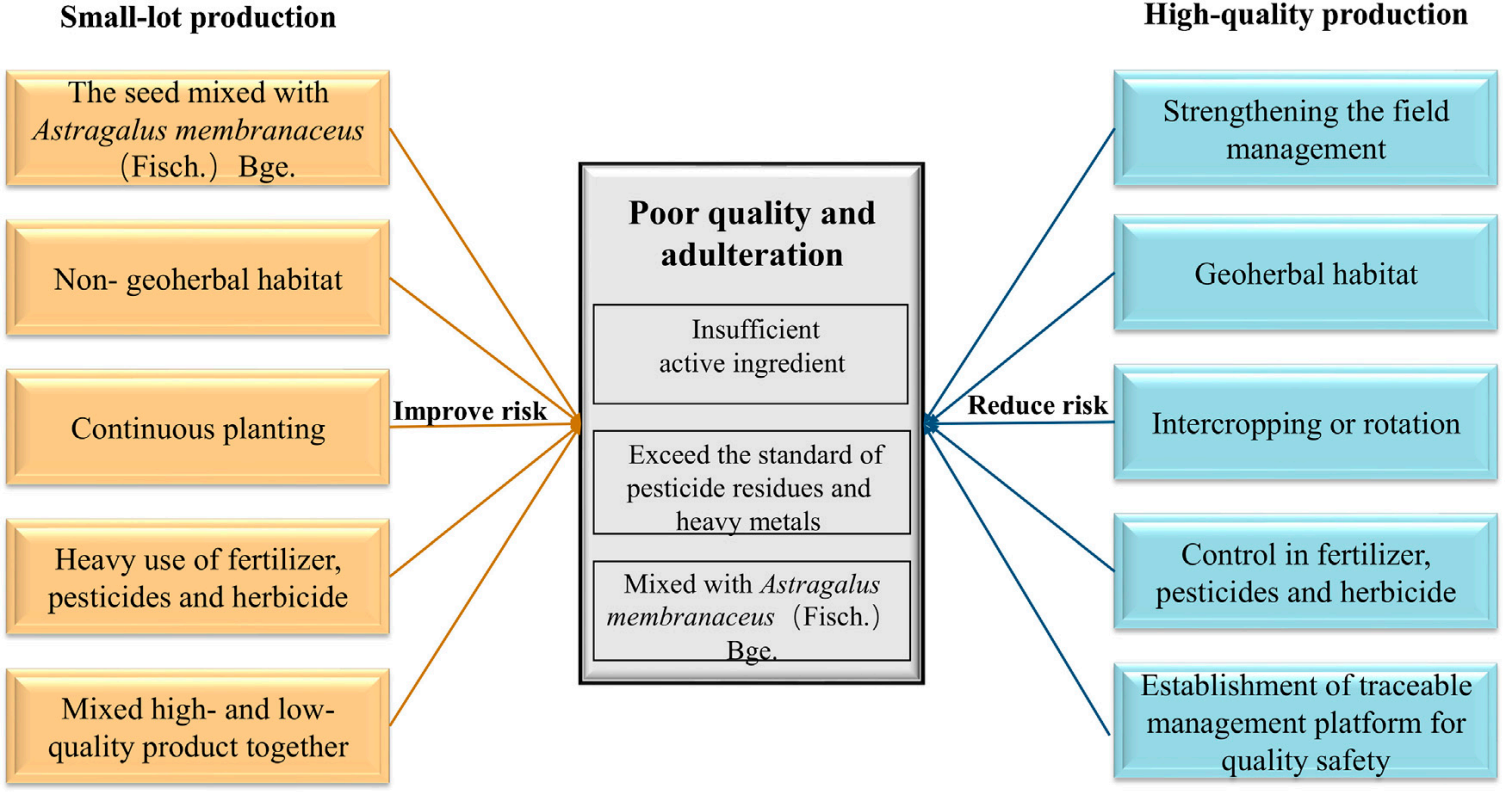
Factors affecting the quality of herbal medicines produced via small-lot and high-quality production models.

At present, due to the introduction of technology, the development trends in RA production are gradually forming into three main markets: traditional markets, high-quality markets, and export markets. The remaining output is generally employed to produce extracts. The different models show large differences in their VCs and behaviors, as illustrated in Figure 7.

Stakeholders in all the VCs will increase their profits in different ways. In traditional markets, growers are more likely to cause quality issues as they take steps to reduce their costs and increase their profits. VCs 1 and 2 are typical examples of traditional markets. In these VCs, in order to achieve a high yield without the help of professional guidance, excessive amounts of chemical drugs may be used, and some unreasonable measures degrade the quality of the RA. Therefore, quality problems are common in these VCs and the RA produced can readily enter the traditional markets but provide relatively low income.

VCs 3–6 lie in between the traditional and high-quality market models. They have a lot of variability available to them and will determine the quality of the medicine according to the market environment.

In high-quality markets, the companies involved participate more completely in some form of self-regulation. As a result, their brand names and reputations improve which raises their overall value. VCs 7 and 8 produce high-quality products that enter the high-quality markets, although the companies involved may not have quality certification. Their production behavior is mainly self-regulated but the reliability and traceability of their products allow them to establish good credibility.

In export markets, the companies involved spend a great deal of time and energy on tracing the quality of the RA from the very beginning (germplasm selection and testing the soil of the planting area) to ensure that their products meet the inspection standards required by the foreign markets.

VC 9 produces high-quality RA as effective quality control measures are implemented during cultivation and production. This brings high financial returns to the stakeholders. Therefore, the producers in high-quality and export markets tend to receive higher incomes from their consumers while traditional market stakeholders receive less.

Selling medicinal materials via the e-commerce platform in VC 10 can greatly reduce store costs. However, there are still many gaps in the supervision process, so there will be some irregular and uncontrolled components. However, high-quality medicinal materials from the same source may also be divided into different levels and circulate into the markets of different channels. Therefore, the behavior and benefits of the stakeholders, as well as the quality of the products and their target markets are all closely related.

According to our survey, the price of RA in traditional markets (50–150 CNY/kg) is lower than that in high-quality or export markets (about 300 CNY/kg). The price of the RA sold ranges from 50 to 550 CNY/kg and it may consist of a mixture of cultivated and wild materials. Moreover, with the improvements made with regard to professional membership of organizations pertinent to traditional Chinese medicine, better guidance is becoming available in different markets. Thus, higher requirements are being placed on the quality of medicinal materials, and the input costs will also be higher. As a result, the price of RA in city hospitals is much greater than that in the Chinese counties. In addition, the price of the RA sold in hospitals is generally greater than that in drug stores, as the products sold in hospitals tend to have a complete chain of quality-inspection links.

## Conclusion

While the increasing demand of RA in the global market has led to the expansion of its production, its supply system is becoming more diverse. One of the purposes of this study is to demonstrate the 10 VCs currently used to produce the RA medicines that are commonly encountered in supplying. Among them, we found that the behavior of stakeholders, the suitability of geographical areas used for cultivation, and the target market all exert critical influences on the quality of RA.

Large-scale stakeholders, such as planting companies are more able to control the development of RA. They are provided with the benefits of large-scale production and quality control to enable quality and stability: they are supported by technology and management structures. On the contrary, small and medium-sized stakeholders are under pressure to invest, and are often too dispersed for good management, and are limited to small-scale production.

The flow of goods and services from producers to consumers matches supply and demand into different markets. Based on morphological characteristics, and active ingredients, the development trends in RA production are gradually forming into three main markets: traditional markets, high-quality markets, and export markets. Besides these, the safe use of herbicides, fertilizers, and pesticides will also affect the choice of target market.

*Daodi* herbs are typical products of geographical origin indication according to good quality and better therapeutic effect in clinical application. The Midwest area of Inner Mongolia and north Shanxi are considered to be suitable for *daodi* RA production. Through the utilization of chemical analysis and a clustering method, *daodi* RA is found to be distinguishable from other products.

During the course of our interviews and quality analyses, some key issues were found in the RA agriculture, processing procedures, and wholesale and retail activities that have largely been overlooked and can reduce the value of the products. Furthermore, we identified the relationship between quality of RA and stakeholder revenues among VCs: these reflected a general trend in the production system. There also seems to be situations in which poor quality and adulteration (intentionally or accidentally) occur due to poor quality control measures in the supply chain.

VCs with vertical integration are found to have more coherent traceability systems. Furthermore, imposing rigorous regulations in the supply chains is beneficial to the implementation of effective quality assurance measures in the manufacturing process. This can efficiently improve the quality and economic value of the final products delivered to consumers. Therefore, there is a strong need to improve the market linkages between producers and customers to create vertically-integrated VCs.

## Data Availability statement

The raw data supporting the conclusions of this article will be made available by the authors, without undue reservation, to any qualified researcher.

## Author Contributions

YB and CZ collected data and wrote the manuscript; ML and RY Initiated the concept and supervised the study; HB, RY, and ML analyzed data and revised the manuscript.

## Funding

This work was supported by Science and technology innovation guidance project in Inner Mongolia (grant No. KCBJ2018040), Science and technology program in Inner Mongolia (grant No. 201701040), Medicine standardization project in Inner Mongolia (grant No. 2018-008), Science and technology achievements transformation project in Inner Mongolia (grant No. CGZH2018174), China Agriculture Research System (grant No. CARS-21), 2019 Chinese medicine public health service subsidy special “the fourth survey on Chinese material medica resource” (grant No. Finance Society (2019) 43), Science and Technology Project of Inner Mongolia Autonomous Region (grant No. 2020GG0144).

## Conflict of Interest

The authors declare that the research was conducted in the absence of any commercial or financial relationships that could be construed as a potential conflict of interest.
