# Supplementary material for: Quality Control of Radix Astragali (The Root of Astragalus membranaceus var. mongholicus) Along Its Value Chains
Source: Front Pharmacol. 2020 Dec 4;11:562376. doi: 10.3389/fphar.2020.562376 (PMC7746871; doi:10.3389/fphar.2020.562376)
Supplement: Supplementary file 1 [file datasheet1.pdf]

Supplementary Material

1 Supplementary Figures

Compound **1**: calycosin-7-glucoside

The reference substance chromatogram of calycosin-7-glucoside (single reference substance) and sample chromatogram of Radix Astragali are shown in **Figure 1**.

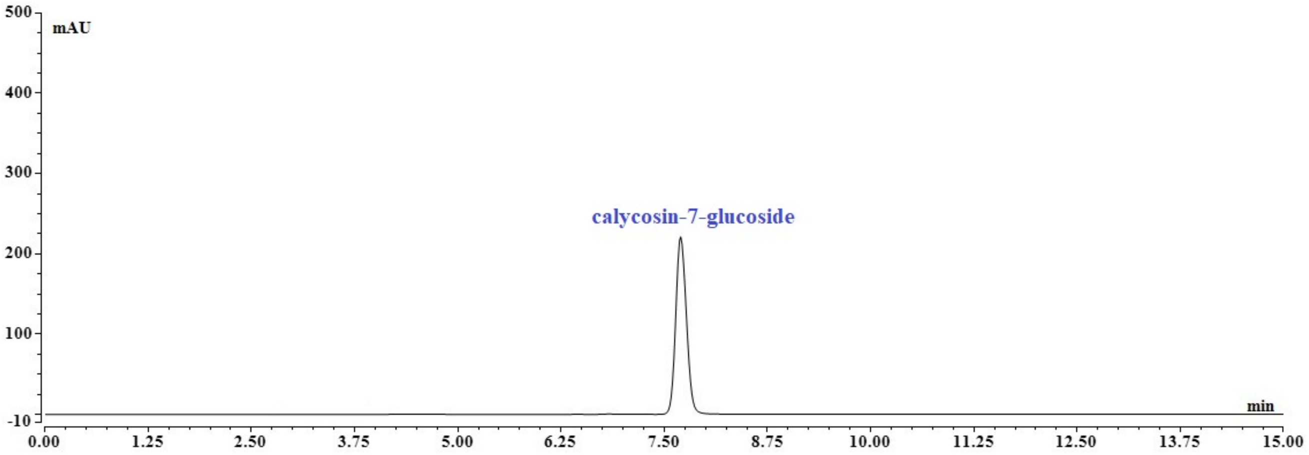

(A)

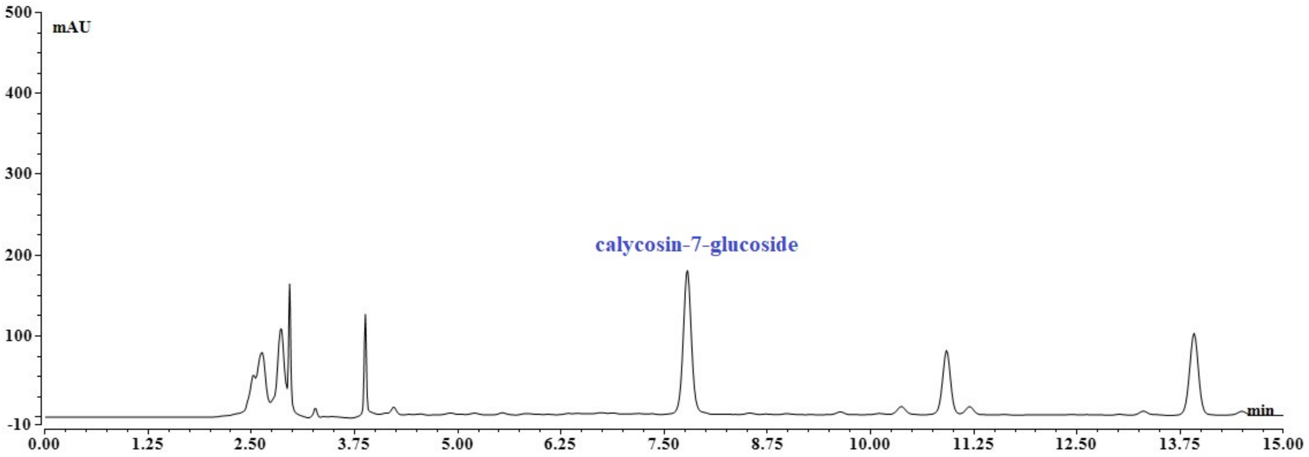

(B)

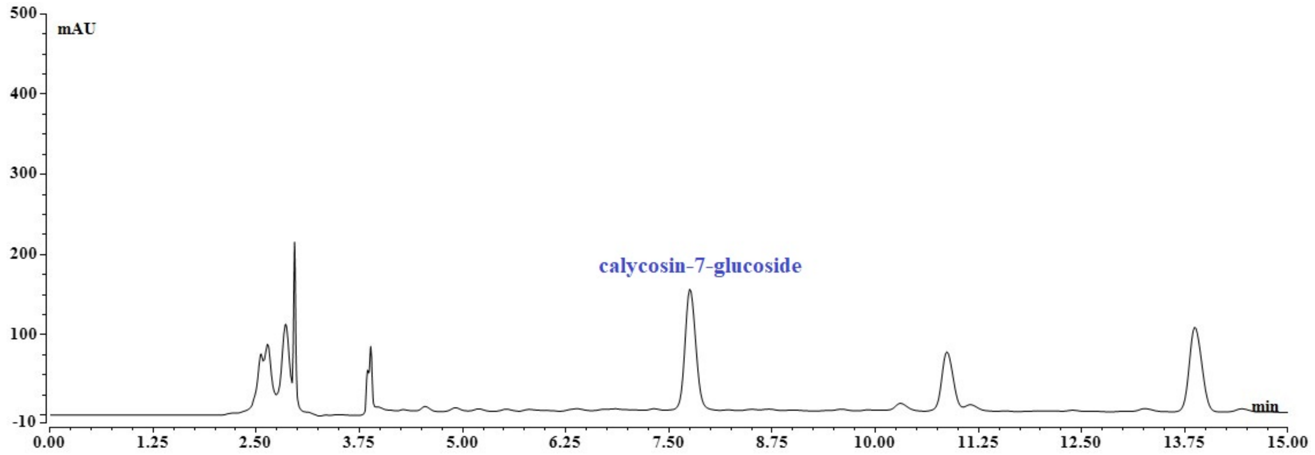
mAU
500
400
300
200
100
-10
calycosin-7-glucoside
min
0.00
1.25
2.50
3.75
5.00
6.25
7.50
8.75
10.00
11.25
12.50
13.75
15.00

(C)

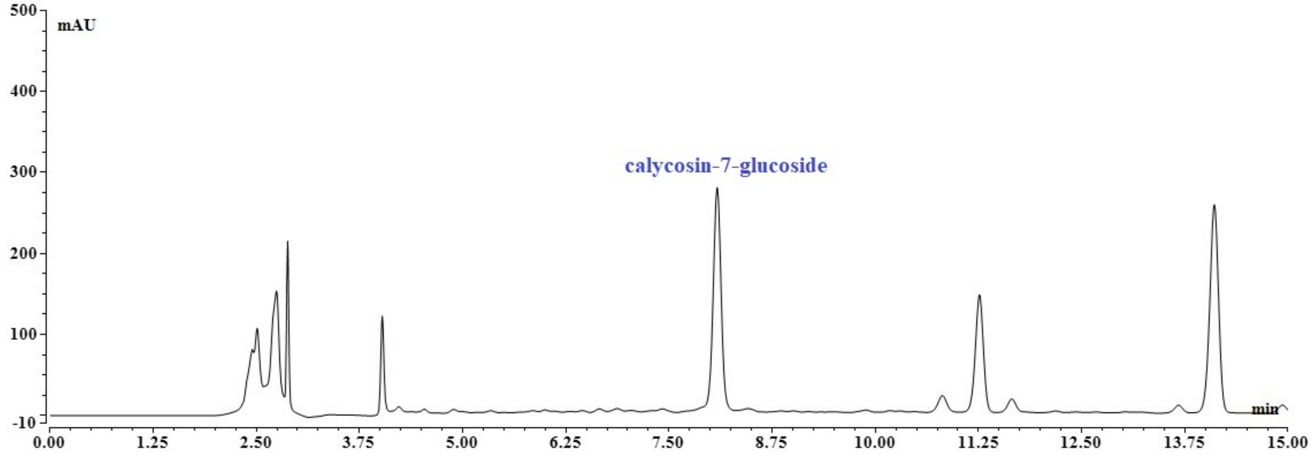
mAU
500
400
300
200
100
-10
calycosin-7-glucoside
min
0.00
1.25
2.50
3.75
5.00
6.25
7.50
8.75
10.00
11.25
12.50
13.75
15.00

(D)

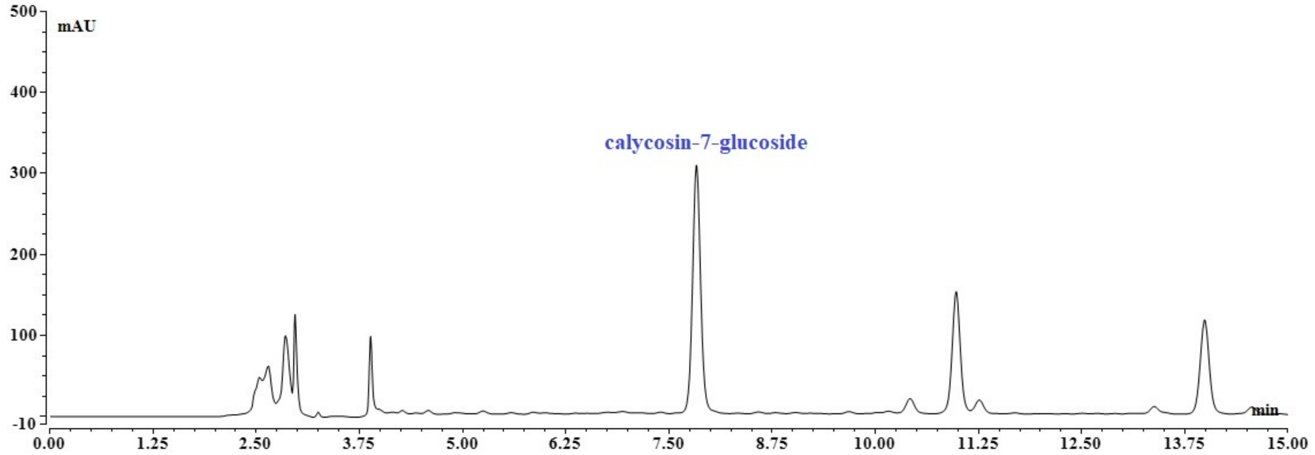
mAU
500
400
300
200
100
-10
calycosin-7-glucoside
min
0.00
1.25
2.50
3.75
5.00
6.25
7.50
8.75
10.00
11.25
12.50
13.75
15.00

(E)

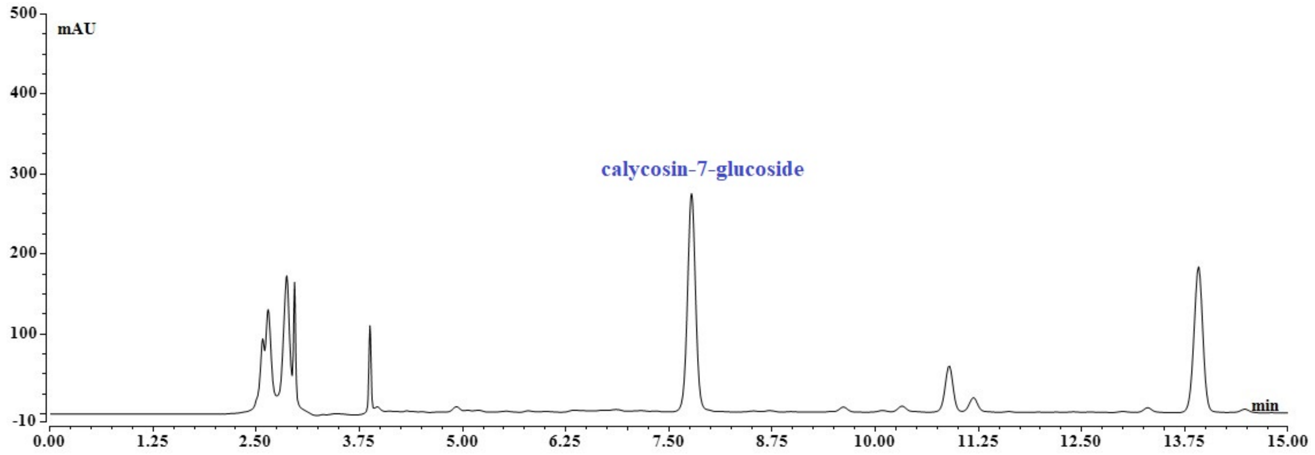

(F)

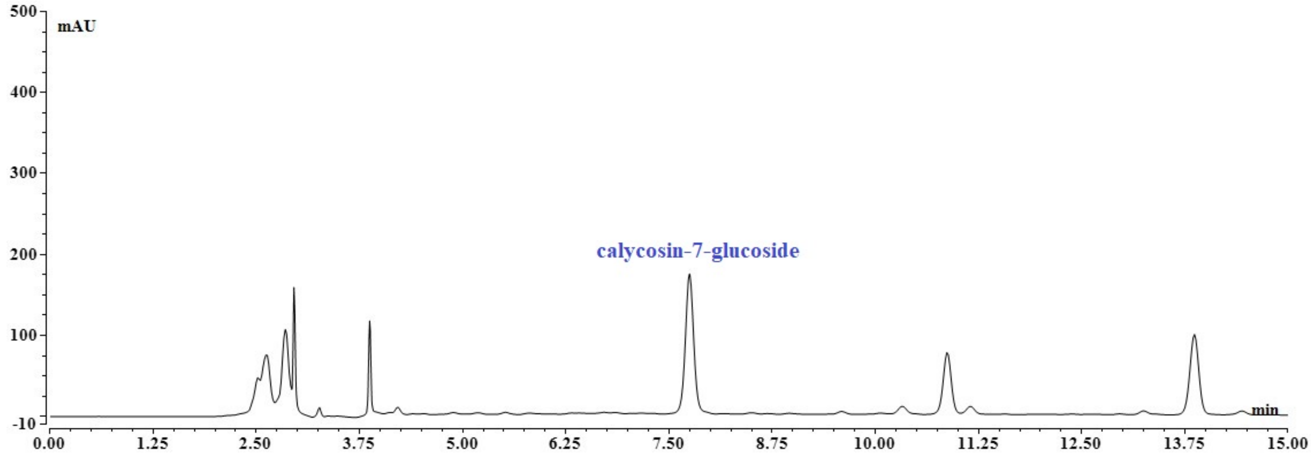

(G)

Supplementary Figure 1 The representative HPLC chromatogram for calycosin-7-glucoside of Radix Astragali

(A) single reference substance; (B) 2019006IM; (C) 2019007IM; (D) 2019011IM; (E) 2019019IM; (F) 2019022IM; (G) 2019025IM.

Compound **2**: astragaloside

The reference substance chromatogram of astragaloside (single reference substance) and sample chromatogram of Radix Astragali are shown in **Figure 2**.

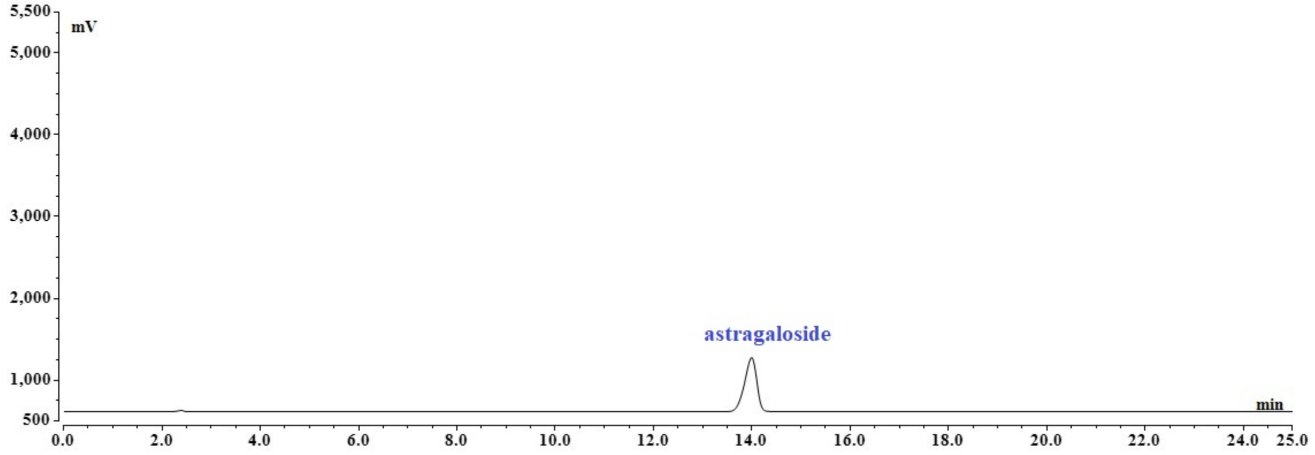
mV
5,500
5,000
4,000
3,000
2,000
1,000
500
astragaloside
min
0.0
2.0
4.0
6.0
8.0
10.0
12.0
14.0
16.0
18.0
20.0
22.0
24.0
25.0

(A)

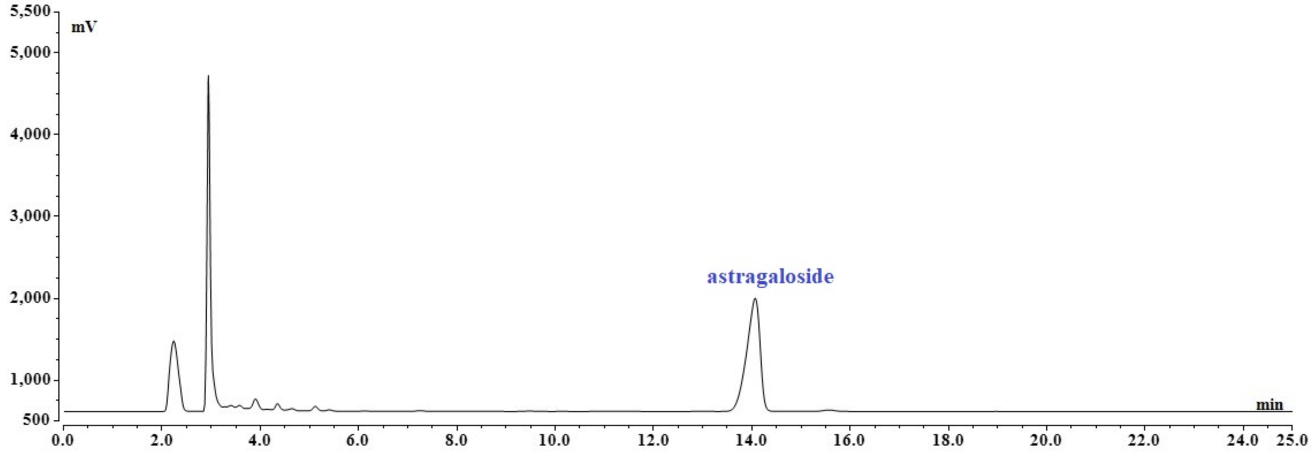
mV
5,500
5,000
4,000
3,000
2,000
1,000
500
astragaloside
min
0.0
2.0
4.0
6.0
8.0
10.0
12.0
14.0
16.0
18.0
20.0
22.0
24.0
25.0

(B)

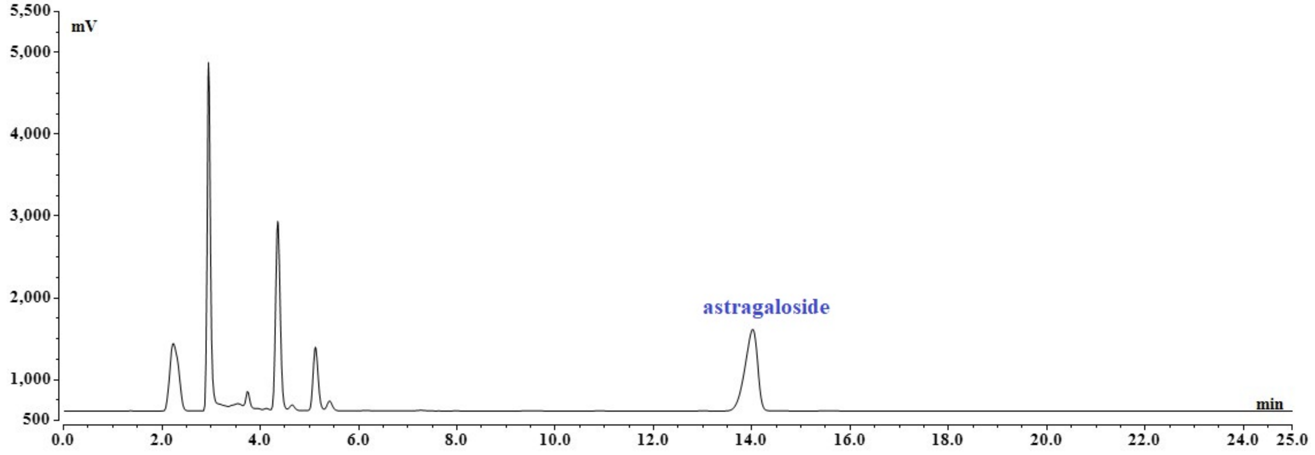
mV
5,500
5,000
4,000
3,000
2,000
1,000
500
astragaloside
min
0.0
2.0
4.0
6.0
8.0
10.0
12.0
14.0
16.0
18.0
20.0
22.0
24.0
25.0

(C)

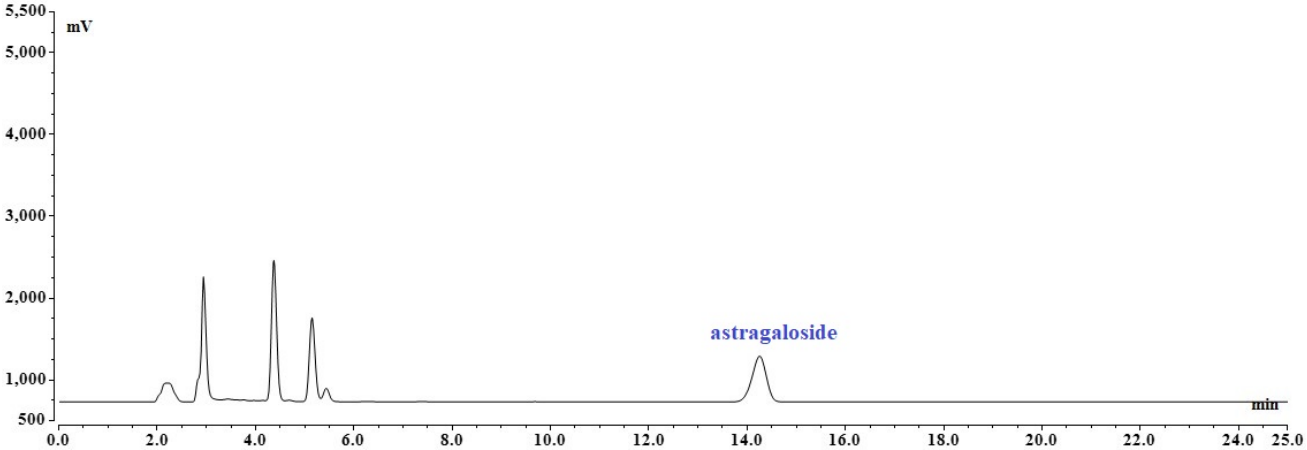
5,500
mV
5,000
4,000
3,000
2,000
1,000
500
astragaloside
min
0.0
2.0
4.0
6.0
8.0
10.0
12.0
14.0
16.0
18.0
20.0
22.0
24.0
25.0

(D)

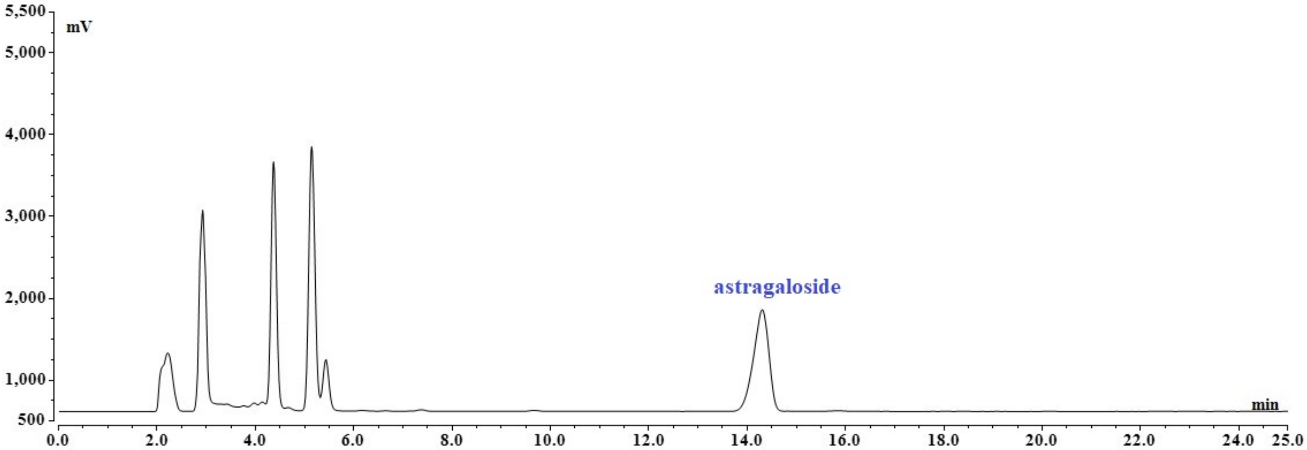
5,500
mV
5,000
4,000
3,000
2,000
1,000
500
astragaloside
min
0.0
2.0
4.0
6.0
8.0
10.0
12.0
14.0
16.0
18.0
20.0
22.0
24.0
25.0

(E)

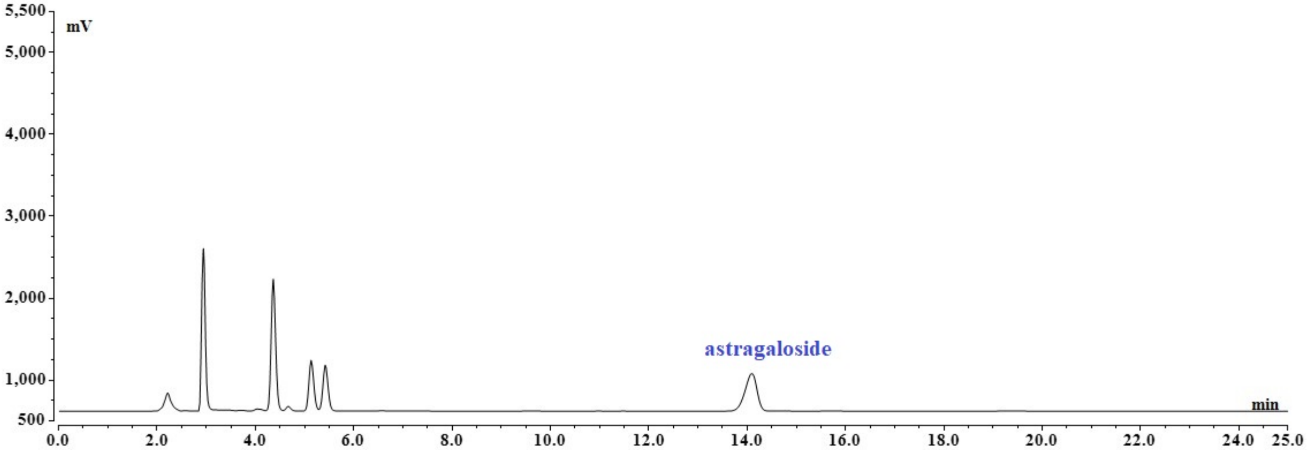
5,500
mV
5,000
4,000
3,000
2,000
1,000
500
astragaloside
min
0.0
2.0
4.0
6.0
8.0
10.0
12.0
14.0
16.0
18.0
20.0
22.0
24.0
25.0

(F)

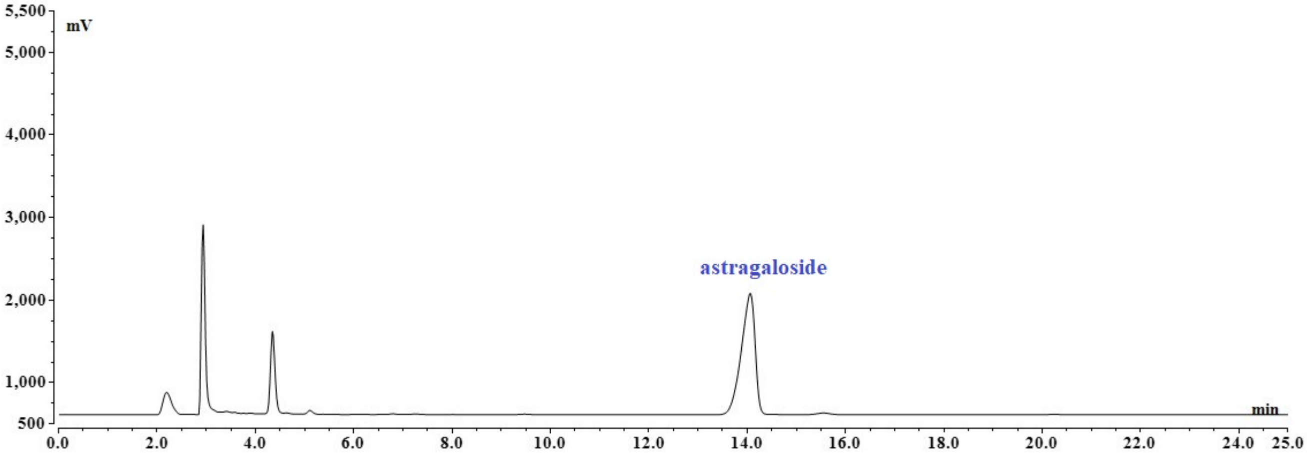

(G)

Supplementary Figure 2 The representative HPLC chromatogram for calycosin-7-glucoside of Radix Astragali

(A) single reference substance; (B) 2019002GS; (C) 2019007IM; (D) 2019012IM; (E) 2019015IM; (F) 2019024IM; (G) 2019031SX.

2 Supplementary Table

Supplementary Table 1 Taxonomic listings.

	**Plant common name**		**Plant full scientific name The Plant List**	**Plant full scientific name Flora of China**
1	Radix Astragali		*Astragalus membranaceus* var. *mongholicus* (Bunge) P.K.Hsiao	*Astragalus membranaceus* var. *mongholicus* (Bunge) P. K. Hsiao
