# Supplementary material for: Quality Control of Radix Astragali (The Root of Astragalus membranaceus var. mongholicus) Along Its Value Chains
Source: Front Pharmacol. 2020 Dec 4;11:562376. doi: 10.3389/fphar.2020.562376 (PMC7746871; doi:10.3389/fphar.2020.562376)
Supplement: Supplementary file 2 [file datasheet2.docx]

Supplementary Material

# Supplementary Table

**The questionnaires to different stakeholders in RA value chains** are shown in **Table 1-3.**

**TABLE 1 Questionnaire (A) for farmers used in fieldwork**

| Occupation | Gender | Ethnicity | Age | Education | level |
| --- | --- | --- | --- | --- | --- |
|  |  |  |  |  |  |
| RA acreage | Mu | | Yield per Mu | Kg | |
| In a cooperative | Y/N | | Other crops reage | Mu | |
| Pesticides and  fertilizer cost | Yuan/Mu | | Amount spent on  land | Yuan/Mu | |
| Primary processing cost | Yuan/Mu | | Pest Control and  Prevention | Personal/Unity measures | |
| Certified grade of quality | Pollution-free/Green/Organical | | Products | Dried/fresh roots | |
| Family size |  | | Number of full labor |  | |
| Monthly living cost | Yuan | | People working on  RA |  | |
| A1. What makes good quality? What do you think is the most important technology for getting a high yield and good quality? | | | | | |
| A2. About Family members: students? The old? Other people’s job? Total income and cost? | | | | | |
| A3. Where are the cultivating technologies from? What species do you plant? Where do you get the seedlings from? | | | | | |
| A4. Pesticides and fertilizer application: Types, quantities, frequency, benefit. Do you know safety use of them? | | | | | |
| A5. What is the most efficient method for primary processing according to your experience? What method do you use? | | | | | |
| A6. Please describe what RA materials can get a high price? Whom do you sell RA to? If the price is not acceptable, what will you do? | | | | | |
| A7. Do you know the price of other RA products? As well as the price of RA in other province? How? Can you use (do you have) smart phone or computer to visit the web? | | | | | |
| A8. Why do you want to start/stop cultivating RA? (For the new/past RA farmer only) | | | | | |
| A9. Are you satisfied with the present production model and why? What would you like to change to improve the situation? | | | | | |

**TABLE 2 Questionnaire (c) for middlemen used in fieldwork**

| Occupation | Gender | Ethnicity | Age | Education level | Year of working |
| --- | --- | --- | --- | --- | --- |
|  |  |  |  |  |  |
| Supply of RA | Kg | | Stock of RA | Kg | |
| Store form |  | | Certified grade of quality | Pollution-free/Green/Organical | |
| Number of product type |  | | Number of staff |  | |
| Cost of the shop |  | | Salary of staff | Yuan/Month | |
| Transportation cost | Yuan | | Packing cost | Yuan | |
| Monthly turnover | Yuan | | Monthly payoff | Yuan | |
| Family size |  | | Number of full labor |  | |
| Monthly living cost | Yuan | | People working on RA |  | |
| A1. What do you think is the most important technology for getting a high yield and good quality? | | | | | |
| A2. About Family members: students? The old? Other people’s job? Total income and cost? | | | | | |
| A3. Please describe the features of good quality RA? Do the good RA have a high price?  What do the consumers most care about RA? | | | | | |
| A4. How do you know the price of RA products? As well as the price of RA in other province? Can you use (do you have) smart phone or computer to visit the web? | | | | | |
| A5. Do you have any suggestion to improve the quality of RA products? | | | | | |
| A6. What kind of RA store will be the most popular in the future? | | | | | |
| A7. To what extend will your income be affected by the fluctuations in RA price? | | | | | |
| A8. Are you satisfied with the present production model and why? Do you want to change something to improve the situation? | | | | | |

**TABLE 3 Questionnaire (D) for retailers used in fieldwork**

| Occupation | Gender | Ethnicity | Age | Education level | Year of working |
| --- | --- | --- | --- | --- | --- |
|  |  |  |  |  |  |
| Supply of RA | Kg | | Stock of RA | Kg | |
| Store form |  | | Certified grade of quality | Pollution-free/Green/Organical | |
| Number of product type |  | | Number of staff |  | |
| Cost of the shop | Yuan | | Salary of staff | Yuan/month | |
| Transportation cost | Yuan | | Packing cost | Yuan | |
| Monthly turnover | Yuan | | Monthly payoff | Yuan | |
| Family size |  | | Number of full labor |  | |
| Monthly living cost | Yuan | | People working on RA |  | |
| A1. What do you think is the most important technology for getting a high yield and good quality? | | | | | |
| A2. About Family members: students? The old? Other people’s job? Total income and cost? | | | | | |
| A3. Please describe the features of good quality RA? Do the good RA have a high price? What do the consumers most care about RA? | | | | | |
| A4. How do you know the price of RA products? As well as the price of RA in other province? Can you use (do you have) smart phone or computer to visit the web? | | | | | |
| A5. Do you have any suggestion to improve the quality of RA products? | | | | | |
| A6. What kind of RA store will be the most popular in the future? | | | | | |
| A7. To what extend will your income be affected by the fluctuations in RA price? | | | | | |
| A8. Are you satisfied with the present production model and why? Do you want to change something to improve the situation? | | | | | |
